# Integrative analyses of bulk microarray data to discover genes, pathways, and immune infiltration characteristics associated with targeting of Ewing sarcoma

**DOI:** 10.1007/s00432-023-04642-0

**Published:** 2023-02-27

**Authors:** Binjie Cao, Haijian Sun, Zhehao Fan, Muhammad Babar Khawar, Liangliang Cai, Shiyi Yu, Zhengyan Liang, Dan Lv, Ning Wang, Caili Bi, Haibo Sun

**Affiliations:** 1 Institute of Translational Medicine, Medical College, Yangzhou University, Yangzhou, China; 2 Jiangsu Key Laboratory of Experimental and, Translational Non-Coding RNA Research, Yangzhou, China; 3 Department of General Surgery, Affiliated Hospital of Nanjing University of Chinese Medicine, Nanjing, China; 4 Applied Molecular Biology and Biomedicine Lab, Department of Zoology, University of Narowal, Narowal, Pakistan

**Keywords:** Ewing sarcoma, Microarray, Immune infiltration, Gene expression, Proliferation

## Abstract

**Purpose:**

To explore transcriptome and immunological features of patients with Ewing sarcoma (ES) using all publicly available microarray data.

**Methods:**

Data of 479 ES tissues were integrated and normalized. Gene expression, immune infiltration, and cancer-specific pathways were analyzed. Genes of interest were knocked down, followed by cell proliferation and colony formation assays.

**Results:**

Consistent with the previous reports of differential expressed genes (DEGs) in ES, our analysis identified *CCND1*, *HMCN1*, and *NKX2-2* were among the most highly expressed, while *TWNC1*, *MYBPC1*, and *CKM* were among the lowest expressed genes. GO, KEGG, and GSEA enrichment analysis identified that the DEGs related to bone and muscle functioning, those that contributed to crucial cellular, and metabolism pathways such as actin binding, apoptosis, TCA cycle, and cell cycle were also significantly enriched. Immune infiltration analysis discovered that many *T* cell subsets including CD4T, CD8 T, and Gamma delta *T* cells were highly infiltrated, while monocytes and B cells were less infiltrated in tumors. A total of 138 genes were both significantly up-regulated in tumors and associated with decreased survival, while 38 significantly down-regulated genes were associated with increased survival, many of which were previously reported as oncogenes and tumor suppressors in ES and other cancers. Silencing of four newly identified top ranked up-regulated genes with decreased survivals in ES inhibited proliferation and colony formation of ES cells.

**Conclusion:**

This study may provide a clear representative transcriptome profile of ES, providing diagnostic biomarkers, pathways, and immune infiltrative characteristics targets for ES.

**Supplementary Information:**

The online version contains supplementary material available at 10.1007/s00432-023-04642-0.

## Introduction

Ewing sarcoma (ES) is a highly malignant bone and soft tissue sarcoma that occurs mostly in children and adolescents (Grünewald et al. 2018). The 5-year overall survival rate has risen to 70–75% in children and adolescents (Whelan et al. 2018; Womer et al. 2012), while adult survival is a little lower than in them (Ahmed et al. 2013). 20–25% of patients had metastases at the time of diagnosis, and they are likely to be resistant to intensive therapy and have shown a very poor cure rate (Gaspar et al. 2015; Khanna et al. 2017).

The disease is characteristic of fusion mutations of the ES gene to an E26 transformation-specific transcription factor, most commonly FLI1 (Riggi et al. 2021). Moreover, many genes such as *LSD1* and some tyrosine kinases have been reported to be overexpressed and considered targets in ES (Balamuth and Womer 2010; Sankar et al. 2014). However, the underlying mechanism of how these driver mutations transform tumorigenesis is still not clear. Thus, a comprehensive understanding of genetic profiles would allow us to uncover crucial differential genes, biomarkers, pathways, and potential therapeutical options for ES patients. However, due to the rare occurrence, resources of genetic studies of ES, especially for RNA-seq data, is limited. Compared with RNA-seq, small cohorts of microarray studies have been reported during the last decades (Agelopoulos 2015; Postel-Vinay et al. 2012; Savola et al. 2011; Scotlandi et al. 2009; Specht et al. 2014; Surdez et al. 2021; Volchenboum et al. 2015), which could be unified and summarized in a representative way. In this study, we performed integrated analyses of all publicly available microarray datasets of ES patients to our best knowledge and uncovered a genetic profile for a better understanding of the characteristics and optional therapeutic targets for ES patients.

## Materials and methods

### Datasets

A total of ten datasets, GSE12102, GSE17679, GSE34620, GSE37371, GSE45544, GSE63157, GSE73166, GSE68776, and GSE142162 (For detailed information, see Table1), attributed to GPL6244, GPL5175, and GPL570 platforms, were obtained from the NCBI database (www.ncbi.nlm.nih.gov/geo/). A total of 20 samples from the GSE37371 dataset, which were tested on the GPL96 platform, were excluded from the study. In total, microarray data of 479 tumor samples and 22 normal muscle samples were applied for subsequent analyses.

**Table 1:** Characteristics of datasets included in the integrated analysis

Platfrom	Accession number	Country	Assay type	Year	PMID
GPL5175	GSE63157	USA	Affymetrix Human Exon 1.0 ST Array	2014	26,052,443
	GSE68776	USA	Affymetrix Human Exon 1.0 ST Array	2015	25,625,846
GPL570 (GPL16311)	GSE12102	Italy	Affymetrix Human Genome U133 Plus 2.0 Array	2008	19,307,502
	GSE17679	Finland	Affymetrix Human Genome U133 Plus 2.0 Array	2009	22,084,725
	GSE34620	France	Affymetrix Human Genome U133 Plus 2.0 Array	2012	22,327,514
	GSE37371	France	Affymetrix Human Genome U133 Plus 2.0 Array	2012	NA
	GSE142162	France	Affymetrix Human Genome U133 Plus 2.0 Array	2021	33,930,311
GPL6244	GSE45544	Germany	Affymetrix Human Genome 1.0 ST Array	2013	2,472,348,626,179,511
	GSE7366	Germany	Affymetrix Human Genome 1.0 ST Array	2016	NA

### Data procession

The Affy package (version 1.68.0) of R software (version 4.0.3) was used to normalize the raw data of datasets (CEL files). According to the microarray annotation file, the expression value of the probe was correlated with the gene expression. We then calculated the average expression level for genes corresponding to multiple probes, sorted and selected the highest value, then combined all the data into one dataset based on overlapping genes.

### Differential gene analysis

Limma package (version 3.46.0) was applied to remove batch effects from the dataset obtained above, and the tumor and normal tissues were compared to identify differentially expressed genes. Genes with adjusted *p* < 0.05 and |logFC|> 1 were deemed to have significantly changed expression.

### Survival analysis

The differentially expressed genes are likely to play important roles in the development of ES. Clinical data for GSE17618 (which is part of GSE17679) matched our hypotheses. Overall survival analysis was performed using the Survival (version 3.2.7) package and Survminer (version 0.4.9) package. The samples (total 44 samples) were split into two groups (high/low) based on the expression of significantly different genes, with a median gene expression level as the cutoff. The Kaplan–Meier survival curves were drawn using the ggplot2 package (version 3.3.3) and log-rank testing for significance. Statistical significance was defined as *p* < 0.05.

### The profile of differentially expressed genes (DEGs)

The heatmap, which displays the expression level of the significant DEGs in samples of the dataset, was created using the pheatmap package (version 1.0.12). The DEGs were displayed using a volcano plot created with the ggplot2 package (version 3.3.3).

### Pathway analysis

The GO database contains three domains: biological process (BP), cellular component (CC), and molecular function (MF). To analyze the function of DEGs, the clusterProfiler package (version 3.18.1) and the KEGG.db package (version 3.2.4) were used to perform GO analysis and KEGG pathway analysis, respectively. The parameter of GO analysis is set as minGSSize = 1, pvalueCutoff = 0.01, qvalueCutoff = 0.01. Gene set enrichment analysis (GSEA) was performed with *h.all.v7.4.symbols.gmt* as a reference gene set. The clusterProfiler Package (version 3.18.1) and enrichplot (version 1.10.2) were used to perform GSEA analysis and visualize the results.

### Immune infiltration analysis

Microarray datasets were uploaded and analyzed in the ImmuCellAI website (http://bioinfo.life.hust.edu.cn/ImmuCellAI#!/analysis) (Miao et al. 2020). A significance test was further performed on the basis of the results of immune infiltration.

### Cell culture

The ES cell line A-673 (RRID:CVCL_0080) was obtained from Procell Life Science Ltd (Wuhan, China). The cell line was maintained in DMEM supplemented with 10% of FBS and 1% of penicillin–streptomycin. Cells were incubated at 37 °C in a humidified atmosphere containing 5% of CO_2_. We declare that the cell line is free of mycoplasma infection and has been identified within 3 years. The testing method is as follows: DNA was extracted by a commercial kit from CORNING (AP-EMN-BL-GDNA-250G). Twenty short tandem repeat (STR) loci plus the gender-determining locus, amelogenin, were amplified by six multiplex PCR and separated on ABI 3730XL Genetic Analyzer. The signals were then analyzed by the software GeneMapper.

### Small interfering RNA transfection

A-673 cells were seeded in six-well culture plates and transfected with siRNA and GP-transfect-Mate (GenePharma, Suzhou, China) according to the manufacturer’s instructions. The culture medium was replaced after 5 h and gene expressions were determined by the reverse transcription‑quantitative polymerase chain reaction (RT‑qPCR) after 2 days. The sequences of all siRNAs tested in the present study are listed in Table S1.

### RT‑qPCR analysis

Total RNA was isolated from ES cell line A-673 using a Tiangen ^®^TRNzol Universal Reagent (TIANGEN Biotech, Beijing, China) as per the manufacturer's protocol. cDNA was reverse transcribed from the total RNA using the HiScript^®^ III RT SuperMix with gDNA wiper for RT‑qPCR (Vazyme Biotech, Nanjing, China) according to the manufacturer’s protocol. RT‑qPCR reactions were performed on an ABI 7900 real‑time PCR system (Thermo Fisher Scientific, Waltham, USA) using the following conditions: 95 ˚C for 30 s, followed by 40 cycles of 95 ˚C for 10 s and 60 ˚C for 30 s. The relative expressions of genes were determined by the 2^−ΔΔCt^ method with GAPDH as an internal reference. The primer sequences applied in the present study are listed in Table S2.

### Cell proliferation assay

A-673 cells were seeded in the six-well culture plates and transfected with siRNAs and GP-transfect-Mate mixtures (GenePharma, Suzhou, China) according to the manufacturer’s instructions. After 48 h, We counted cells directly under the microscope as the starting cell count. Then we counted the number of cells at 72 h, 96 h and 120 h, respectively. Finally, the proliferation rate relative to the number of starting cells was calculated.

### Colony-forming cell assay

A-673 cells were seeded in the six-well culture plates and transfected with siRNAs and GP-transfect-Mate mixtures (GenePharma, Suzhou, China) according to the manufacturer’s instructions. The culture medium was replaced after 5 h. cells were then harvested after 1 day, and 500 of them were reseeded on 6-cm plates with DMEM complete medium for approximately 14 days of incubation. Colonies were finally fixed with methanol, stained with crystal violet dye, and counted using ImageJ software.

### Prognosis analysis of single-sample geneset group

The samples were clustered using the R package mclust (version 5.4.10) based on the most significant gene set from GSEABase (http://www.gsea-msigdb.org/gsea/msigdb/human/geneset/HALLMARK_E2F_TARGETS.html), and variations in the gene expression of different clusters were presented using heatmaps. Based on these differences, the samples (total 44 samples) were classified into two groups. The R package survival (version 3.2.7) and Survminer (version 0.4.9) were used to perform univariate COX regression analysis on different groups to detect the degree of prognostic impacts.

## Results

### Study overview and dataset characteristics

**Fig. 1:**
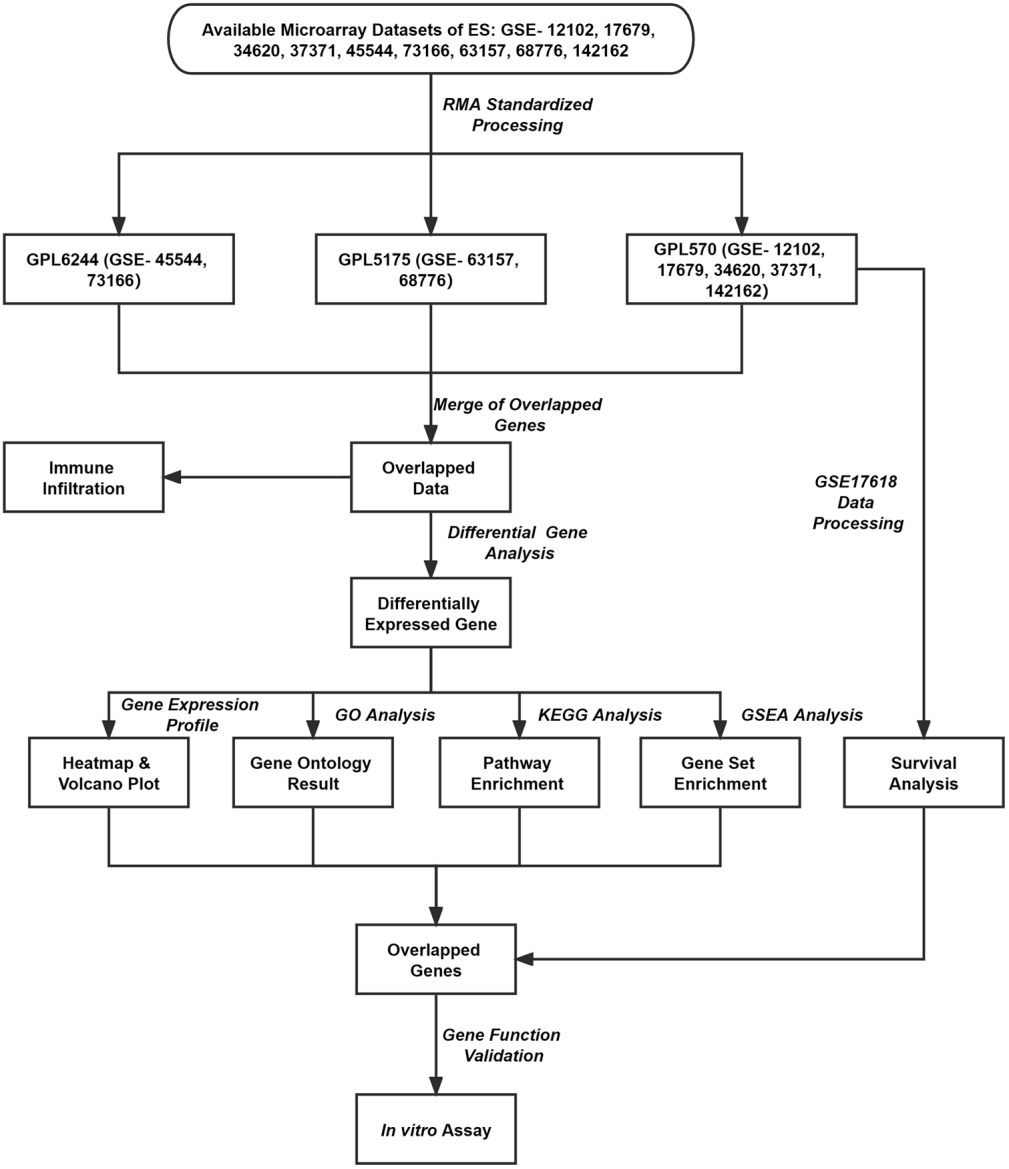
Study workflow shows overall steps carried out in the integrated microarray analyses

As shown in Fig. 1, step-by-step analyses were carried out using the integrated microarray dataset derived from nine independent microarray studies, covering 479 ES samples and 22 muscle samples. Prognostic impact (Kaplan–Meier) analysis of DEGs was done on a sub-cohort (GSE17618) where the survival data is available.

### Identification of DEGs in ES

**Fig. 2:**
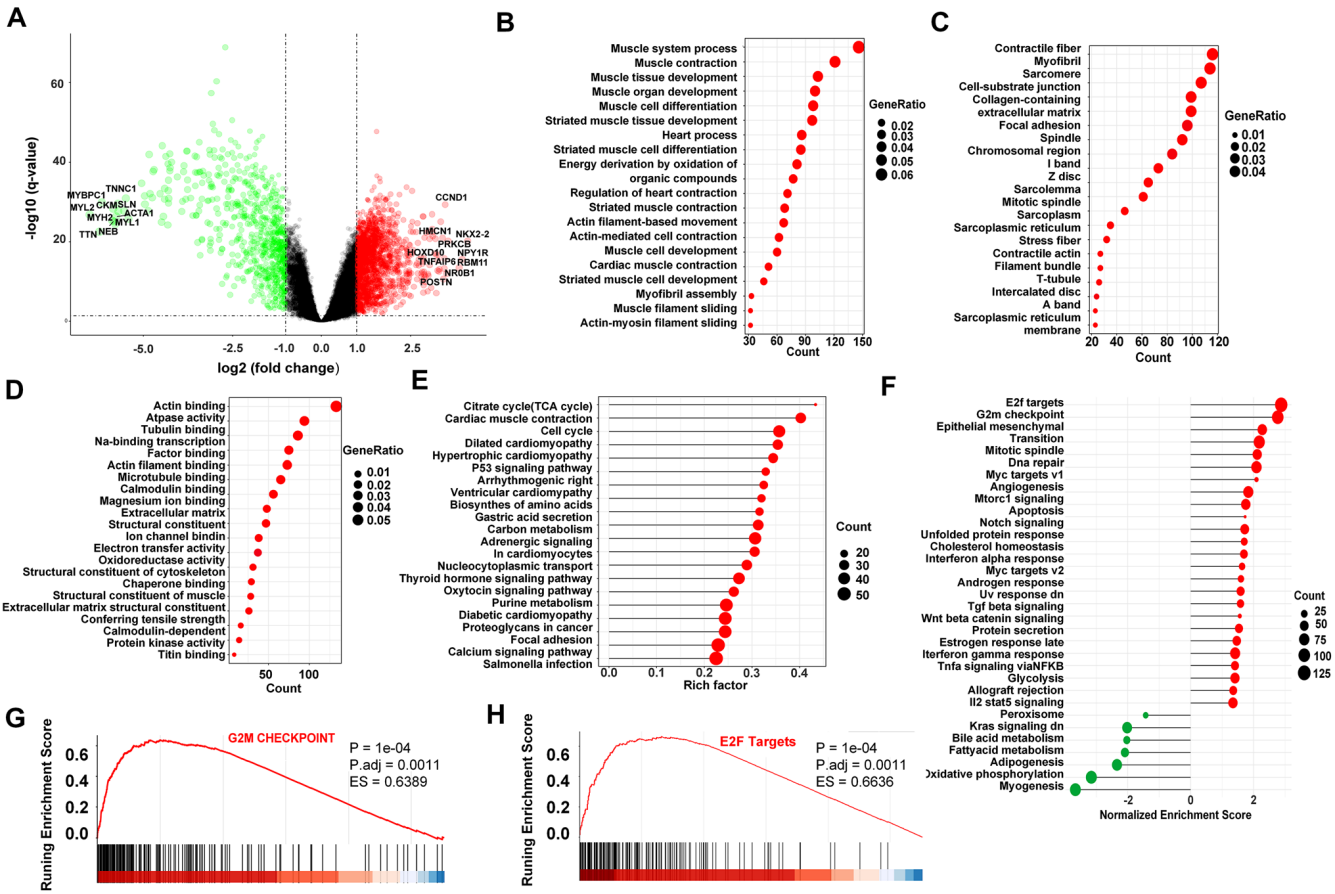
Identification of differentially expressed genes (DEGs) and enriched pathways in ES. (**A**) A volcano plot of DEGs in tumor samples compared to normal samples. Red indicates the gene expression was up-regulated in tumor samples compared to normal samples (*P* < 0.05 and logFC > 1); green indicates the gene expression was down-regulated in tumor samples compared with normal samples (*P* < 0.05 and logFC < -1); Black indicates the |logFC|< 1. (**B**–**D**) GO analysis. The size of the dots represents the gene ratio. **E** KEGG pathway enrichment analysis. The size of the dots represents the number of DEGs in the pathway. **F** GSEA analysis. Red indicates NES > 0; green indicates NES < 0. The size of the dots represents the number of DEGs in the pathway. (**G**–**H**) The GSEA plot of the two pathways: G2M checkpoint and E2F Targets

After a proper normalization of batch effects among different arrays (Fig. S1), we observed a total of 2383 DEGs (Table S3), including 1601 up-regulated and 782 down-regulated genes, using the *P* value < 0.05 and |logFC|> 1 criterion in ES samples compared to normal muscle samples, with green and red representing low and high expression levels, respectively (Fig. S4A). Similarly, a volcano plot of DEGs was depicted and the top ten logFC genes were listed (Fig. 2A). To our interest, several genes on our list have been reported to be involved in the progression of ES, such as *NKX2-2*, *HMCN1*, *HOXD10*, and *PRKCB*. *NRX2-2* participates in the formation of the core regulatory circuitry, activates EWS–FLI1 transcription, and significantly promotes the proliferation of ES cells both in vitro and in vivo (Sarver et al. 2015; Shi et al. 2020; Surdez et al. 2012; Svoboda et al. 2014). To our interest, the DEGs were overwhelmingly attributed to the development of muscle and bone, indicating that the disease interrupts muscle and bone growth, which is also evidenced by plain radiographs exhibiting permeative and infiltrative destruction of the affected bone (mainly in the diaphysis of the long bone) (Ozaki 2015). Similarly, intramyofiber invasion, a particular phenomenon in which malignant cells exhibit intracellular spread into skeletal muscle cells, usually occurs in ES (Mahjoub et al. 2006).

### Identification of enriched pathways in ES

The GO database categorizes gene and protein functions into three categories: biological process (BP), cellular component (CC), and molecular function (MF). The top 20 significant categories in BP, CC, and MF are shown Fig. 2B–D. The DEGs were primarily involved in the muscle-related processes in the BP category such as muscle system, muscle contraction, and muscle tissue development. Contractile fibers, myofibrils, sarcomere-related genes, and those associated with the growth of muscles showed considerably higher expression in CC. Furthermore, the MF analysis revealed an abundance of actin, actin filament, and calmodulin binding-related genes. The genes related to the bone and muscle functioning also showed higher expression. The DEGs were largely associated with the development of muscle and bone, suggesting that the development of Ewing sarcoma impacts muscle and bone growth. In addition, KEGG pathway enrichment analysis discovered that the DEGs were found to be engaged in several crucial cellular pathways associated with cancer development and cell activity, such as the p53 signaling pathway and the cell cycle (Fig. 2E). We also found that several non-cancer related pathways including citrate cycle, dilated cardiomyopathy, cardiac muscle contraction and focal adhesion were enriched in KEGG analysis. Interestingly, many of these pathways such as dilated cardiomyopathy and cardiac muscle contraction, have also been discovered to be related to ES progress in a recent study (Ding et al. 2021). Finally, GSEA analysis showed 25 significantly activated and 7 suppressed pathways (Fig. 2F). The top two activated pathways (NES > 0) were E2F targets and G2M checkpoint, both of which were related to the cell cycle, indicating that DEGs have possible effects on the cell cycle (Figs. 2G–H) and many other altered gene sets involved in such as KRAS, apoptosis, and DNA repair pathways (Figs. S2-3). In addition, seven suppressed pathways including oxidative phosphorylation were enriched by GSEA analysis. Indeed, ES is a highly aggressive and metabolically active malignant tumor and its metabolic activity can broadly be characterized by features of glycolytic activity and oxidative phosphorylation (Atreyi Dasgupta and Shuck 2017). A previous study has also shown that strong oxidative phosphorylation metabolism is associated with a well-defined range of EWSR1-FLI1 activity (Aynaud et al. 2020).

### Analysis of association between DEGs and survival

**Fig. 3:**
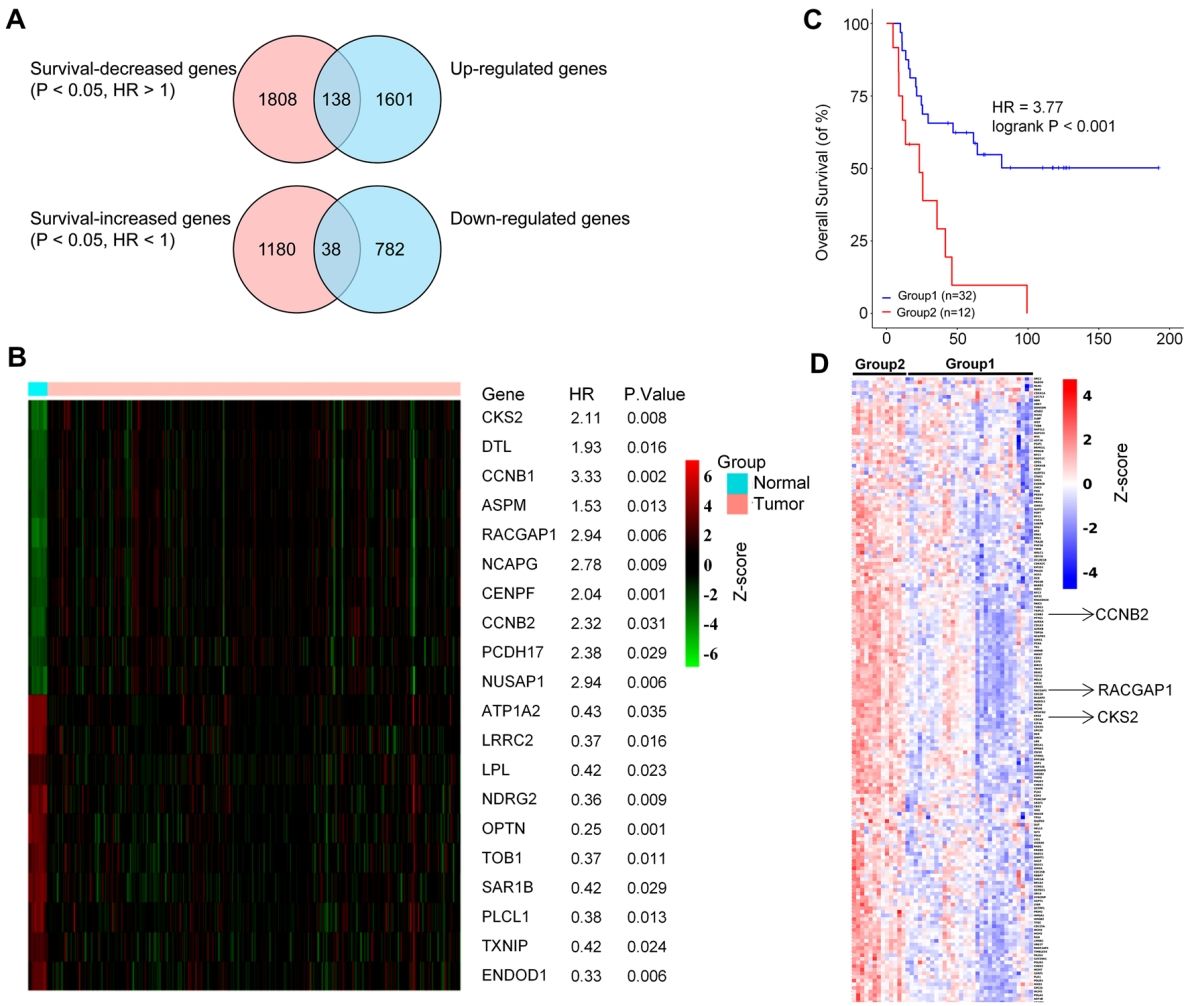
Analysis of association between DEGs and survival. (**A**) Screening of DEGs. A total of 138 overlapping DEGs were identified in the two datasets which satisfied the criteria HR > 1 and logFC > 1. A total of 38 overlapping DEGs were identified in the two datasets which satisfied the criteria HR < 1 and logFC < -1. **B** Heatmap of the top ten overlapping DEGs (10 + 10) mentioned above. **C** The samples were grouped based on the expression level of the E2F-Targets geneset, and survival analysis was performed using GSE17618 survival information. The red and blue lines represent two different groups. **D** Gene expression heatmap of E2F-Targets geneset for grouped samples

The up-regulated and down-regulated genes were screened using the logFC > 1 or logFC < -1 criteria. Then GSE17618 datasets were used to determine the survival. A total of 138 overlapping DEGs were identified in the two datasets which satisfied the criteria *P* value < 0.05, HR > 1, and logFC > 1. Similarly, a total of 38 overlapping DEGs were identified in the two datasets, which satisfied the criteria *P* value < 0.05, HR < 1 and logFC < -1 (Fig. 3A), and a heat map of the of all the overlapping DEGs (Fig. S4B) and the top 10 up-regulated and down-regulated overlapping DEGs and their prognostic impacts are shown (Fig. 3B). In addition, we selected the most significant pathway gene sets in the GSEA analysis to validate the association of pathways with prognosis. As shown in Fig. 3C, enrichment of E2F-Targets pathway-based gene set grouping showed decreased overall survival in our dataset. Many previously identified DEGs including *CCNB2*, *RACGAP1*, and *CKS2* also participated in this pathway (Fig. 3D). Together, these results demonstrated that E2F-Targets pathway may play an important role in the progression of ES.

### Immune infiltration analysis

**Fig. 4:**
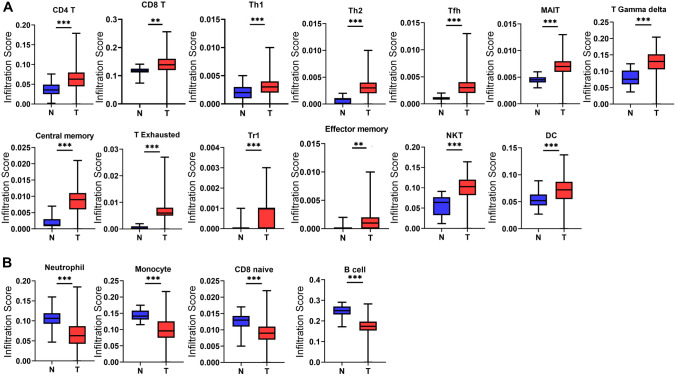
Immune infiltration analysis results. Significant highly (**A**) and lowly (**B**) immune infiltrated lymphocytes in tumor than those in normal samples are shown

We performed the immune infiltration analysis between tumor and normal samples. We calculated the proportion of each immune infiltrated cell type (Fig. S5) and observed that a number of *T* cell subgroups, such as CD4 T, CD8 T, Gamma delta T, Tcm, and Tex, were highly infiltrated in tumor samples than those in normal samples. On the contrary, larger amounts of monocytes and B cells were infiltrated in normal samples than in tumor samples (Fig. 4A, B), while other lymphocytes were not significantly altered (Fig. S6).

### In vitro verification of four up-regulated DEGs with decreased survival

**Fig. 5:**
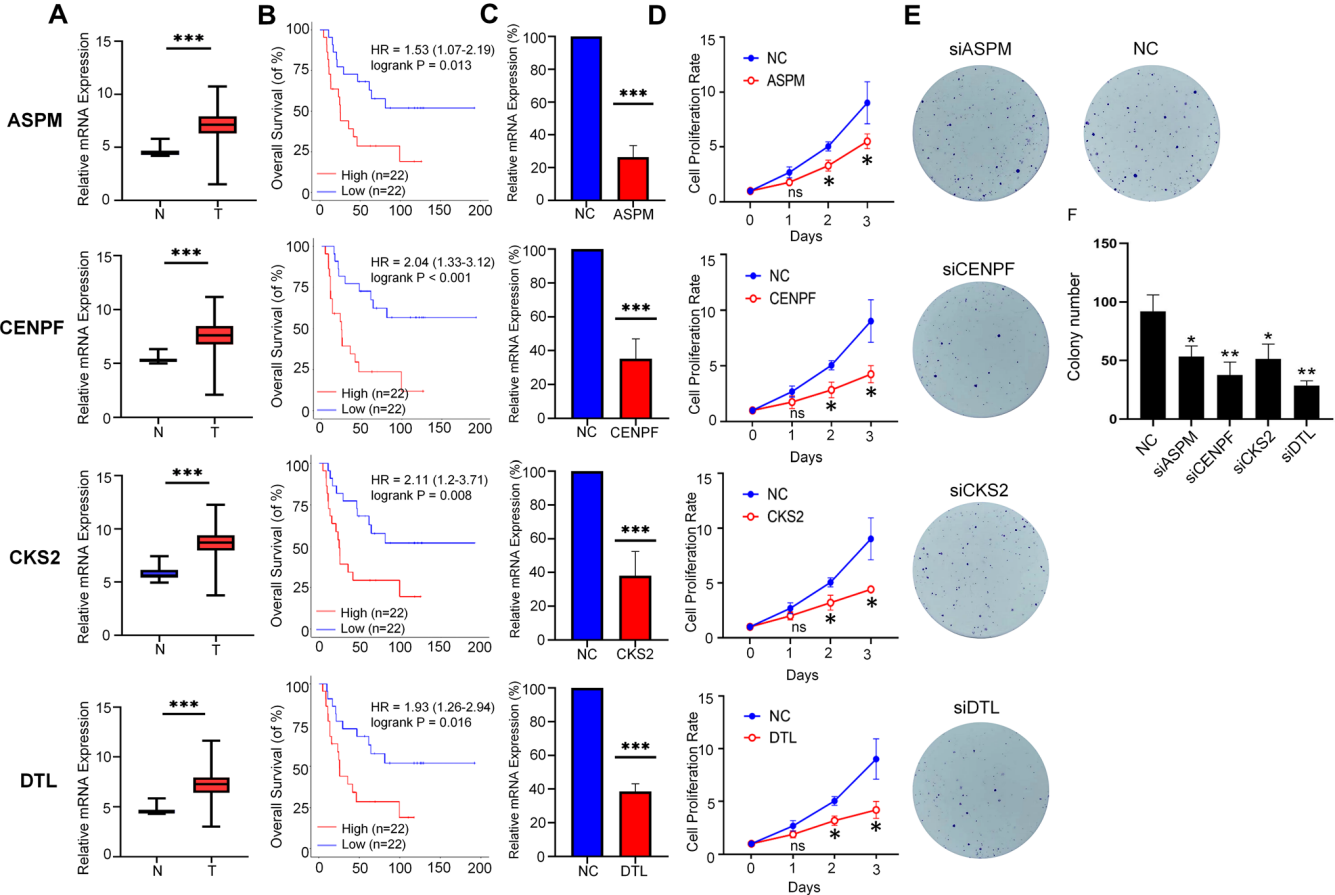
In vitro verification of four up-regulated DEGs with decreased survival. (**A**) Comparison of expression of four DEGs (*ASPM*, *CENPF*, *CKS2*, and *DTL*) between tumor and normal samples. Normal samples (N): blue, tumor samples (T): red. **B** The Kaplan–Meier survival curves of the selected four genes. **C** RT‑qPCR verification of silencing efficiencis of siRNAs targeting the respective four genes. (**D**) Cell proliferation assay tesing knock-down effects of the four genes. A-673 cells were cultured in six-well plates and transfected with siRNAs, respectively. Cell counts were performed on day 1, day 2 and day 3. Data are shown as mean ± SD, *n* = 3 independent experiments. (**E**–**F**) Clonogenic assay testing knock-down effects of the four genes. 500 cells with transfection of respective siRNAs were transferred to six-well plates and the cell culture was maintained for 14 days for colony formation observation. **E** Representative images of cell colonies. **F** Quantitative results of colony-forming assay. Data (Fig. 5A) are shown as mean ± SD, *n* = 22 (normal) v.s. *n* = 479 (tumor). Data (Fig. 5**C**, **D**, **F**) are shown as mean ± SD, *n* = 3 independent experiments. (ns: *p* > 0.05, *: *p* < 0.05, **: *p* < 0.01, ***: *p* < 0.001)

To further confirm and evaluate potential therapeutic values from the novel identified up-regulated DEGs with the impacts of decreased survival in the current study**,** we chose to validate genes that were the top ten up-regulated DEGs with decreased survival and not shown to be associated with tumorigenesis. Among them, studies have proved that *CCNB1*, *RACGAP1* and *NUSAP1* promote the progression of multiple cancers (Aljohani et al. 2022; Gu et al. 2022; Guo et al. 2020), and *NCAPG* and *CCNB2* have been found to be associated with a poor prognosis of ES (Kakar et al. 2022; Zhao et al. 2022), while *ASPM*, *CENPF*, *CKS2*, and *DTL* have not been studied in ES to the best of our knowledge. Interestingly, these four genes were also found in the GO analysis and GSEA results (Fig. 2B–H). As shown in Fig. 5A, B, increased expressions of these four genes are significantly linked to worse overall survival. We then transiently transfected siRNAs targeting *ASPM*, *CENPF*, *CKS2*, and *DTL*, and the confirmed high knock-down efficiencies by qRT-PCR in A-673 cells (Fig. 5C). Direct cell counting under microscope after transfection revealed that all knock-down experimental groups had significantly fewer cells on days 2 and 3 compared to the siRNA control group (Fig. 5D). Furthermore, clonogenic assay demonstrated that silencing of the four genes severely reduced the ability of A673 cells to form colonies (Fig. 5E, F).

## Discussion

Due to its rare occurrence, ES lacks a thorough transcriptome study in contrast to those cancer types that have been extensively sequenced and profiled. Here, we undertook an integrative analysis of publicly accessible microarray datasets of ES with the intention of creating a comprehensive transcriptome profile of ES for a better understanding of its features and identifying specific therapy alternatives. Consistent with previous microarray studies (Fagone et al. 2015), we showed cyclin D1 to be the highest expressed gene in ES through the current integrated microarray database analysis (Magro et al. 2015). Cyclin D1 has been shown to be frequently up-regulated in many different cancer types and its up-regulation triggers tumorigenesis through induction of transcription, translation, and protein stability (Kim and Diehl 2009). We analyzed the role of cyclin D1 in ES through pathway enrichment analysis, and found that E2F targets and G2/M checkpoints were the two most highly enriched pathways. This is consistent with previously discovered functions of cyclin D1, although its precise role in ES is still unclear. Our results suggest that the overexpression of E2F-cyclin D1 co-stimulating regulatory loops can enhance cell cycle progression, potentially leading to uncontrolled tumor cell growth (Alao 2007; Schulze et al. 1994). Our analysis has also shown a positive prognostic role of the E2F-Targets pathway. Therefore, Cyclin D1 may also be employed as a molecular diagnostic biomarker in ES. Interestingly, another ES variant, the BCOR–CCNB3 fusion-positive sarcoma, has also been shown to up-regulate CCND1 (Kao et al. 2018). On the basis of the successes of CDK4/6 inhibitors in dedifferentiated liposarcoma (Lin et al. 2020), as well as the current clinical data supporting the use of CDK4/6 inhibitors in subsets of sarcoma primarily driven by CDK4/6 deregulation (Hsu et al. 2022), available CDK4/6 small molecule inhibitors targeting the E2F pathway may be promising for the treatment of variants of ES including the BCOR–CCNB3 fusion subset.

*NKX2-2* is another among the highest expressed genes in our analysis. To our interest, *NKX2-2* has been exclusively studied in ES and is recognized as a specific biomarker of this malignancy. *NKX2-2* is a major target gene and a core co-regulatory member of the driver mutation fusion gene, *EWS–FLI1* in ES (Shi et al. 2020; Smith et al. 2006). Our study again confirms *NKX2-2* as a valuable biomarker for ES.

Up-regulation of the *ASPM* gene has recently been shown and recognized as a poor outcome factor in glioma (Chen et al. 2020) and liver malignancies (Lin et al. 2008). *CKS2* is recognized as a tumor promoter that functions as a regulator of the gene translation of numerous validated targets including p53, *CDK1*,cyclin A, caspase-3, and *Bax* (You et al. 2015). CENPF is a microtubule-binding protein that has been found to be associated with poor prognosis in several types of cancer (Sun et al. 2019). However, the mechanism behind this correlation remains unclear. *DTL*, an E3 ubiquitin-protein ligase, has also been shown a poor prognostic role to promote cancer progression in several cancer types (Cui et al. 2019; Luo et al. 2022). On the other hand, the present study identified *NDRG2* and *TOB1* as down-regulated DEGs with prolonged survival in ES. *NDRG2* has been shown to inhibit the occurrence and metastasis of tumors and increase the sensitivity of anti-cancer drugs. Additionally, the functions of *NDRG2* as a tumor suppressor contribute to tumor growth inhibition and anti-metastasis in various tumors (Lee et al. 2022). Like many other tumor suppressor proteins, down-regulated *TOB1* expression has been reported in various cancers, mostly in breast, pancreas, thyroid, and stomach. *TOB1* expression levels are inversely associated with the tumorigenicity and metastatic ability of breast cancer cell lines as well as with tumor progression in patients with breast cancers (Lee et al. 2015). To the best of our knowledge, we present here for the first time evidence of up-regulation and poor prognostic outcomes associated with four genes in ES. These findings underscore the importance of conducting further in-depth studies of these genes in the future.

Most sarcomas including ES are considered “cold” cancers based on their low tumor mutation burden (TMB) and low immunogenicity (Rytlewski et al. 2021). Although short of experimental proof, our immune infiltration results indicate ES may still be able to stimulate an immune response, evidenced by increased immune infiltration of many types of lymphocytes including memory and effector T cells, NKT cells, and DC cells. Thus, CAR- or immune checkpoint inhibitors-based immunotherapy may still be promising therapeutic options for ES patients.

## Conclusion

Our findings provided a clearer representative transcriptome profile of ES. These uncovered prognostics-associated genes, pathways, and immune infiltrative characteristics might be valuable for future study.

## Supplementary Information

Below is the link to the electronic supplementary material.

Supplementary file1 (TIF 125282 KB)

Supplementary file2 (TIF 125282 KB)

Supplementary file3 (TIF 125282 KB)

Supplementary file4 (TIF 125282 KB)

Supplementary file5 (TIF 125282 KB)

Supplementary file6 (TIF 125282 KB)

Supplementary file7 (TIF 125282 KB)

Supplementary file8 (TIF 125282 KB)

Supplementary file9 (XLSX 12 KB)

Supplementary file10 (XLSX 10 KB)

Supplementary file11 (XLSX 248 KB)

## Abbreviations

ES: Ewing sarcoma
GEO: Gene Expression Omnibus
DEGs: Differentially expressed genes
GO: Gene ontology
KEGG: Kyoto Encyclopedia of Genes and Genomes
CC: Cellular component
MF: Molecular function
BP: Biological process
GSEA: Gene set enrichment analysis
NC: Negative control

## Declarations

### Data availability

A total of ten datasets, GSE12102, GSE17679, GSE34620, GSE37371, GSE45544, GSE63157, GSE73166, GSE68776, and GSE142162, were obtained from the NCBI database (www.ncbi.nlm.nih.gov/geo/).
